# Ehlers-Danlos syndrome type IV

**DOI:** 10.1186/1750-1172-2-32

**Published:** 2007-07-19

**Authors:** Dominique P Germain

**Affiliations:** 1 Centre de référence pour la maladie de Fabry et les maladies héréditaires du tissu conjonctif (syndromes d'Ehlers-Danlos, pseudoxanthome élastique, mucopolysaccharidoses), Assistance Publique – Hôpitaux de Paris, Paris, France

## Abstract

Ehlers-Danlos syndrome type IV, the vascular type of Ehlers-Danlos syndromes (EDS), is an inherited connective tissue disorder defined by characteristic facial features (acrogeria) in most patients, translucent skin with highly visible subcutaneous vessels on the trunk and lower back, easy bruising, and severe arterial, digestive and uterine complications, which are rarely, if at all, observed in the other forms of EDS. The estimated prevalence for all EDS varies between 1/10,000 and 1/25,000, EDS type IV representing approximately 5 to 10% of cases. The vascular complications may affect all anatomical areas, with a tendency toward arteries of large and medium diameter. Dissections of the vertebral arteries and the carotids in their extra- and intra-cranial segments (carotid-cavernous fistulae) are typical. There is a high risk of recurrent colonic perforations. Pregnancy increases the likelihood of a uterine or vascular rupture. EDS type IV is inherited as an autosomal dominant trait that is caused by mutations in the *COL3A1 *gene coding for type III procollagen. Diagnosis is based on clinical signs, non-invasive imaging, and the identification of a mutation of the *COL3A1 *gene. In childhood, coagulation disorders and Silverman's syndrome are the main differential diagnoses; in adulthood, the differential diagnosis includes other Ehlers-Danlos syndromes, Marfan syndrome and Loeys-Dietz syndrome. Prenatal diagnosis can be considered in families where the mutation is known. Choriocentesis or amniocentesis, however, may entail risk for the pregnant woman. In the absence of specific treatment for EDS type IV, medical intervention should be focused on symptomatic treatment and prophylactic measures. Arterial, digestive or uterine complications require immediate hospitalisation, observation in an intensive care unit. Invasive imaging techniques are contraindicated. Conservative approach is usually recommended when caring for a vascular complication in a patient suffering from EDS type IV. Surgery may, however, be required urgently to treat potentially fatal complications.

## Disease name and synonyms

Ehlers-Danlos syndrome type IV

Sack-Barabas syndrome

Vascular Ehlers-Danlos syndrome

Vascular type of Ehlers-Danlos syndromes (EDS)

## Definition

Ehlers-Danlos syndrome type IV, also known as the vascular type of Ehlers-Danlos syndromes (EDS), is an inherited disorder of connective tissue characterised by severe arterial and digestive complications which are rarely, if at all, observed in the other forms EDS [1,2]. Patients with EDS type IV, most of whom display characteristic facial features and premature ageing of limb extremities (acrogeria), are predisposed to vascular and digestive ruptures, as well as perforations of the gravid uterus. Arterial ruptures account for the majority of deaths, whilst digestive perforations, occurring mainly on the sigmoid colon, are less often fatal. These complications, rare in childhood, affect 25% of patients before the age of 20, and 80% by the age of 40 [1]. The median age of death is estimated to be 50 years. Due to different clinical symptoms, natural history and prognosis, EDS type IV should be assessed separately within the group of EDS.

## Epidemiology

The Ehlers-Danlos syndromes are a group of hereditary disorders of connective tissue, whose prevalence is estimated between 1/10,000 and 1/25,000, with no ethnic predisposition. The Villefranche classification identifies six clinical types (Table 1) [3], among which the vascular EDS (OMIM #130050) accounts for about 5 to10% of cases [4].

**Table 1:** Classification of Ehlers-Danlos syndromes

**Type**	**Former nosology**	**OMIM #**	**Inheritance**	**Gene and locus**	**References**
Classical type	Type I	130000	AD^a^	*COL5A1*, 9q34 *COL5A2*, 17q21 Other?	[59, 60]
Classical type	Type II	130010	AD	*COL5A1*, 9q34 *COL5A2*17q21 Other?	[59, 60]
Ehlers-Danlos like syndrome with Tenascin X deficiency		606408	AR^b^	*TNXB*, 6p21.3	[61] [62]
Hypermobility type	Type III	130020	AD	*TNXB*, 6p21.3 Other?	[63]
Vascular type	Type VIA Type VIB	225400	AR	*PLOD*, 1p36 ?	[64]
Arthrochalasia type	Types VIIA and VIIB	130060	AD	*COL1A1*, 17q21 *COL1A2*, 7q22	[65] [66]
Dermatosparaxis type	Type VIIC	225410	AR	*ADAMTS2*, 5q23	[67]
Progeroid type		130070	AD	*XGPT1*, 5q35	[68]
Periodontitis type	Type VIII	130080	AD	?, 12p13	[69]
Ehlers-Danlos variant with periventricular heterotopia		300537	XL^c^	*FLNA*, Xq28	[70]

^a^AD: Autosomal dominant

^b^AR: Autosomal resessive

^c^XL: X-linked

## Clinical presentation

Clinical diagnosis of vascular Ehlers-Danlos syndrome is based on four criteria: a characteristic facial aspect (acrogeria) in most patients, thin and translucent skin with highly visible subcutaneous vessels, ecchymoses and haematomas, and arterial, digestive and obstetrical complications.

### A. Facial dysmorphy

When present, *acrogeria *is defined by characteristic facial features such an emaciated face with prominent cheekbones and sunken cheeks. The eyes appear sunken or bulging, often with colouring around them and thin telangiectasia on the eyelids [5]. The nose is pinched and thin, as are the lips, particularly the upper lip whose edges are undefined [5]. A non-acrogeric form of the syndrome may also exist, whose clinical diagnosis is more difficult as several distinguishing features are not present [6].

### B. Skin symptoms

In vascular EDS, the skin is abnormally thin and pale. It is smooth, soft and velvety. The veins under the skin are distinctly visible as the skin is translucent on the thorax, the shoulders and, sometimes, the abdomen. The skin on the extremities appears prematurely aged, hence the term acrogeria, and the subcutaneous veins are highly visible. We have recently shown that anteflexion of the trunk during clinical examination reveals highly visible subcutaneous vessels on the lower back. In our experience, this sign has proved highly valuable in establishing the clinical diagnosis [Germain DP, unpublished data].

However, there is no hyperelasticity of the skin in EDS type IV, in contrast to classical EDS (types I and II) and hypermobile EDS (type III) [7]. Fragility of the skin may be observed, though less often than in classical EDS. This leads to wounds with an abnormally long scarring process. Secondary enlargement of scars and deposits of residual haemosiderin are typical.

### C. Ecchymoses and haematomas

**Figure 1:**
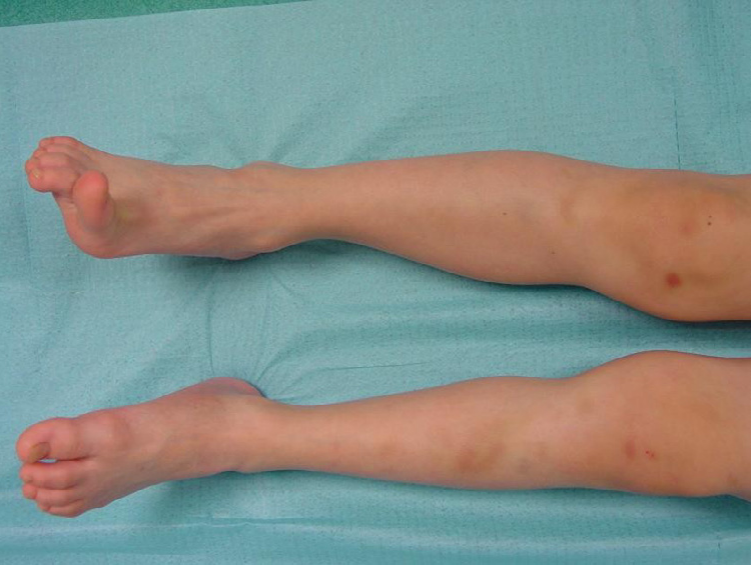
Extensive bruising of the legs in a child affected with Ehlers-Danlos syndrome type IV.

Ecchymoses and haematomas are common [6] and extensive bruising (Figure 1) is one of the major diagnostic criteria in the Villefranche nosology of Ehlers-Danlos syndromes [3].

### D. Complications

Patients suffering from vascular EDS are prone to arterial, digestive and obstetrical complications.

#### 1) Vascular complications

The exact nature of the vascular lesions is disputed in the literature, as most of these correspond to arterial dissections or tears caused by the deterioration of congenitally thin and fragile tissue, leading to haematomas, false aneurysms or intracavitary bleeding. Most of the aneurysms recorded in the literature are probably 'false aneurysms', even though a percentage of patients display real fusiform aneurysms [8]. Arterial ruptures or dissections are responsible for the majority of deaths as they are unpredictable [9] and because the fragility of arterial walls often makes the surgical repair difficult [10-13].

All anatomical areas can be affected, with a tendency toward arteries of large and medium calibre [14]. The disease frequently involves the proximal branches of the aortic arch, the descending thoracic aorta and the abdominal aorta. The distal branches of the aorta, especially the renal, mesenteric, iliac and femoral arteries, are also particularly affected [10,15-17].

**Figure 2:**
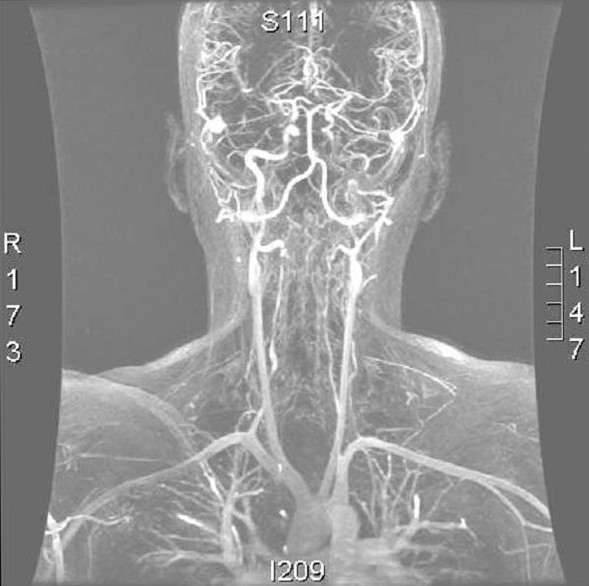
Angio-MRI (coronal section) of the supra-aortic vessels after injection of 20 cc of gadolinium in a woman aged 24 with EDS type IV, revealing a dissecting haematoma of the left internal carotid (black arrow), a bilateral dissection of the vertebral arteries in their V1 and V2 segments (white arrows) and a dissection of the middle and distal third of the right subclavian artery (head of arrow).

**Figure 3:**
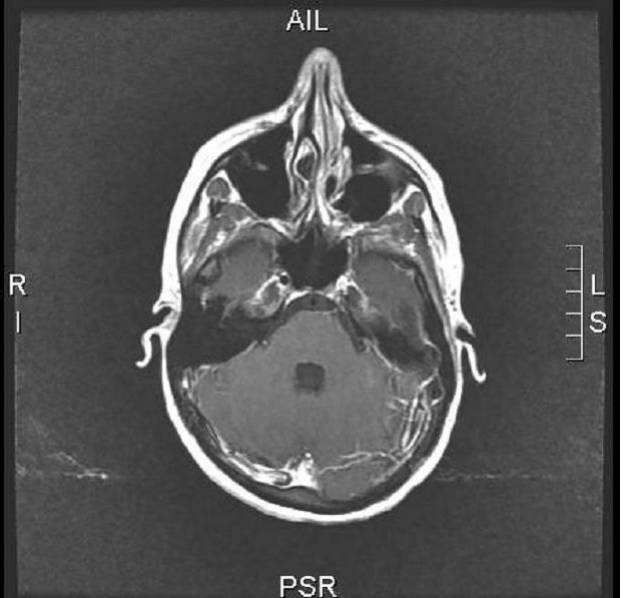
Cerebral angio-MRI after injection of 20 cc of gadolinium in a woman aged 24 with EDS type IV displaying a dissecting haematoma of the left internal carotid.

Dissections of the vertebral arteries and the carotids in their extra- and intra-cranial segments have been widely documented and are a typical complication of the syndrome (Figures 2, 3) [18-20]. Carotid-cavernous fistulae (CCF) are another typical complication of EDS type IV [21,22] due to reduced content of collagen III in the arterial walls. CCF clinical diagnosis is based on the existence of tinnitus, thrill, headaches and pulsating exophthalmos [16,18,19].

EDS type IV should therefore be considered after any ischemic stroke in young subject [5,23]. In addition, intracranial haemorrhages are found in 4% of cases, half of which are caused by the rupture of a previously-identified intracranial aneurysm [20]. This prevalence appears higher than in the general population, where the frequency of unruptured aneurysms is estimated between 0.5 and 1% [19]. Early diagnosis of brain haemorrhage in patients with EDS type IV is important, as it has significant implications for the care of patients and their relatives. In patients with confirmed diagnosis of vascular EDS, non-invasive techniques (echo-Doppler, angioscan, angio-MRI) are absolutely imperative for diagnosing arterial dissections or aneurysms [24]. Arteriograms are associated with a high rate of complications at the point of puncture and/or tear of the arterial wall and are thus contraindicated [25]. The benefit/risk ratio of any invasive diagnostic procedure should be carefully assessed and arteriogram reserved only for cases where arterial embolisation is planned [24]. If thrombosis occurs after carotid or vertebral dissection, anticoagulation may be required but should be carried out with care. Surgical treatment of brain aneurysms has a high morbidity mortality rate owing to the brittle nature of the tissue in these patients. Endovascular radiology treatments also have a high post-treatment morbidity and mortality [22,24].

The low instance of cerebrovascular complications does not work in favour of systematic scanning for brain aneurysms in asymptomatic patients suffering from EDS type IV, in whom the risks linked to surgery often contraindicate surgery before the appearance of symptoms [19].

There is some controversy about the usefulness of serial vascular check-up. Some experts advise against them because of the anxiety that the discovery of aneurysmal lesions (for which a treatment decision would rarely be taken) may provoke in the patients. Many authors, though, recommend carrying out an annual or biennial check-up including an ultrasound scan of the supra-aortic vessels and the arterial axes of the lower limbs, and a thoracic-abdominal scan with careful, low pressure injection [24]. The discovery of a previously unknown large or rapidly-expanding aneurysm requires close monitoring. For a fortuitously-discovered vascular lesion which threatens the vital prognosis, the planned surgery is thus appropriate outside of an emergency context, even though post-operative treatments are often complicated by haemorrhages or tears of the anastomoses [8].

#### 2) Digestive complications

**Figure 4:**
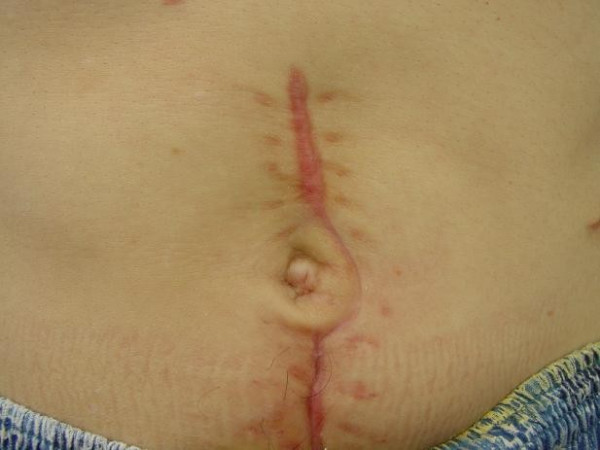
Surgical laparotomy scar following sigmoid colon perforation in a young patient presenting with vascular Ehlers-Danlos syndrome.

Most perforations occur in the sigmoid colon [26] but the small intestine can occasionally be affected (Germain DP, unpublished data). Spontaneous ruptures of the spleen and the liver have also been described [27]. There is a high risk (50%) of multiple colonic perforations and leakage from the anastomosis in case of simple segmental resection with immediate re-establishment of continuity [12,28]. The treatment of choice is therefore partial colectomy with colostomy, possibly followed by secondary re-establishment of continuity (Figure 4). Alternatively, total colectomy with ileostomy and closure of the rectal stump or ileo-rectal anastomosis may be proposed despite the young age of patients, because of the risk of recurrent colonic perforations and the scarcity of perforations of the small intestine [16]. There is a significant risk of leakage on the anastomosis.

Mortality due to digestive perforations in patients suffering from vascular EDS is relatively low, estimated at 2% [1] and, therefore, lower than some descriptions from isolated clinical cases may indicate [29].

#### 3) Obstetrical complications

Pregnancy can increase the likelihood of a uterine or vascular rupture in women suffering from EDS type IV (particularly during the last three months) [30]. Maternal mortality stands at around 12% [1,5,31]. The highest is the risk during labour, delivery and immediate post-partum period. Uterine haemorrhages occur frequently during the post-partum period and are sometimes only treatable by hysterectomy. The value of a caesarean carried out before the onset of labour (in order to minimise the risks related to contractions and take better control of haemostasis) [32] has not yet been the subject of a controlled study [24]. The prophylactic use of desmopressin to control primary haemostasis has been proposed [33].

#### 4) Pleuropulmonary complications

Pneumothoraces, haemoptysis [34] and haemorrhagic cavitary lesions [35] of the pulmonary parenchyma have occurred in several patients suffering from EDS type IV [6].

#### 5) Mitral valve prolapse

An increased frequency of mitral valve prolapse (MVP) has been reported in patients with EDS type IV [36]. However, it should be noted that this is the case in many inherited disorders of connective tissue and that the exact prevalence of MVP in the general population is unknown [7].

## Aetiology

### Genetics

#### Mode of transmission

Vascular EDS is caused by heterozygote mutations of the *COL3A1 *gene and is transmitted as an autosomal dominant trait [1,5,7,37].

#### Gene location

Type III collagen is coded by an unique gene, *COL3A1*, whose locus is situated on the long arm of chromosome 2, in position 2q24.3-q31 [38]. Linkage analyses have demonstrated that vascular EDS co-segregates with polymorphic markers in this locus [39]. There is no genetic heterogeneity and the polymorphic markers in the *COL3A1 *locus can occasionally allow the allele associated with the disease to be identified in families and can prove useful for indirect molecular diagnosis.

### Physiopathology

Collagens are a family of proteins that contribute to the organisation of the extracellular matrix, comprising at least 19 proteins coded by at least 35 non-allelic genes dispersed in the genome [40]. EDS type IV is caused by a deficit of type III collagen, which belongs to the fibrillar collagens. All fibrillar collagens are homo- or heterotrimerics formed by the linking of three monomers or **α **chains. Type III collagen is a homotrimeric formed by the linking of three **α**1(III) chains, with the central part of the molecule adopting a triple-helix structure. The amino acid sequence of the triple helix is characterised by repeated glycine-X-Y sequences, where X and Y are often the amino acids proline and hydroxyproline respectively. In order to ensure correct linking of **α **monomers, there should be no interruption in the repetition of the glycine-X-Y triplets and the length of the triple helix should remain similar for each **α **chain [5].

Type III collagen is a constituent of arterial walls. Its quantitative or qualitative deficit in EDS type IV accounts for the propensity of arterial tears or dissections which characterise this illness. The walls of the digestive tract are also rich in type III collagen, which explains why digestive perforations are another frequent complication of EDS type IV [41,42].

## Diagnosis

### Clinical criteria (Table 2)

**Table 2:** Vascular Ehlers-Danlos syndrome: Villefranche diagnostic criteria (adapted from [3]

**Major diagnosis criteria**	Arterial, digestive or uterine fragility or rupture
	Thin, translucent skin
	Extensive bruising
	Characteristic facial appearance
**Minor diagnosis criteria**	Positive family history, sudden death in a close relative
	Acrogeria
	Hypermobility of small joints
	Tendon and muscle rupture
	Talipes equinovarus (clubfoot)
	Early onset varicose veins
	Spontaneous pneumothorax or haemothorax

Diagnosis of EDS type IV is mainly clinical and is easier when the patient is acrogeric, has a positive family history or has displayed a first instance of arterial or digestive complication.

### Laboratory diagnosis

#### 1) Biochemical diagnosis

**Figure 5:**
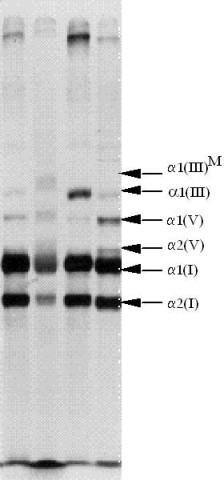
Sodium dodecyl sulfate-PolyAcrylamide Gel Electrophoresis (SDS-PAGE) analysis of collagens secretion in a patient affected with vascular EDS and a control. The band corresponding to type III collagen is indicated by **α**1(III). Columns 1 and 3 correspond to the supernatant culture medium of the cutaneous fibroblasts. Colums 2 and 4 correspond to cell extracts. Secretion of type III collagen in the medium is reduced in the patient (column 1) as compared to the control (column 3). Conversely, there is intracellular retention of abnormal collagen in the cell extracts of the patient (column 2) as compared to the control. In addition, the mutant collagen III has a higher molecular weight due to additional post-translational modification **α**1(III)^M^.

The study of the secretion of collagen III by skin fibroblasts may demonstrate a quantitative or qualitative deficit with abnormal migration of the pro**α**1(III) chains to electrophoresis of proteins on polyacrylamide denaturant gel (*Sodium Dodecyl Sulfate-Polyacrylamide Gel Electrophoresis*, SDS-PAGE) (Figure 5) [37].

#### 2) Molecular biology

**Figure 6:**
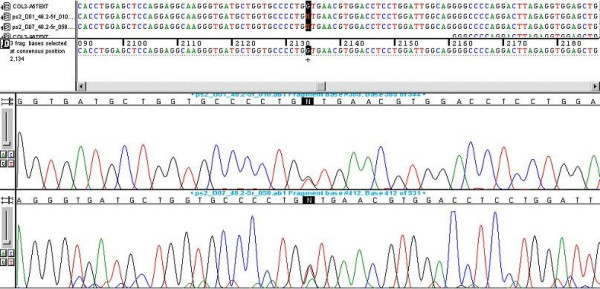
Detection of a heterozygote missense mutation (G514V) in the *COL3A1 *gene in a 47-year old female patient affected with Ehlers-Danlos syndrome type IV. A G to A substitution was found at nucleotide position 2042 starting from the initiation codon (ATG) of the *COL3A1 *gene. This nucleotide substitution alters the codon (GGT) for glycine to the codon (GTT) for valine at position 514 of the **α**-chain of collagen type III protein. Two electrophoregrams of the same patient are shown. (Figure courtesy of Prof. X. Jeunemaitre)

The publication of the complementary DNA (cDNA) sequence of the *COL3A1 *gene has paved the way towards understanding of the molecular basis of EDS type IV [43-45]. Direct molecular analysis, which is difficult because of the large size of the gene and the major allelic heterogeneity, allows the mutation of the *COL3A1 *gene to be characterised exactly (Figure 6). EDS type IV can be caused by missense point mutations affecting the glycine residues of the triple helix [46], splicing mutations with exon skipping [47], small or large deletions [44] or haploinsuffiency [48]. Each mutation is particular to a given family [5,49].

#### 3) Histology

##### Optical microscopy

The observation of a skin biopsy *via *optical microscopy does not usually contribute a great deal but can occasionally reveal a thinned dermis, within which the groups of collagen appear sparse and/or irregular [5].

##### Electron microscopy

Ultrastructure study can sometimes reveal images of dilation of the granular endoplasmic reticulum of the skin fibroblasts, irregularities in the diameter of collagen fibres and an unidentified fibrino-granular substance within the extracellular matrix. Given the high number of false negatives, the absence of these images should not exclude the diagnosis EDS type IV [5].

#### 4) Haemostasis

Haemostasis tests are normal with the possible exception of an increased bleeding time in some patients. The tendency toward haemorrhages in EDS type IV therefore appears to be due to fragility of the tissue and the capillaries, rather than a thrombocytic or plasmatic defect [50].

## Differential diagnosis

In childhood, coagulation disorders and Silverman's syndrome are the most often-cited differential diagnoses, owing to the propensity for haematomas and ecchymoses in EDS type IV. In adulthood, the other Ehlers-Danlos syndromes, as well as Marfan syndrome (OMIM #154700) and Loeys-Dietz syndrome (OMIM #609192) caused by mutations in the genes *TGFR1 *(OMIM #190181) or *TGFR2 *(OMIM #190182) [51,52], and arterial tortuosity syndrome (OMIM 208050) caused by a deficit in GLUT10 [53] can sometimes pose a problem. Similarly, when no mutation of the *COL3A1 *gene has been identified, the possible existence of yet unidentified hereditary disorders of connective tissue causing arterial aneurysms or dissections, and therefore mimicking EDS type IV, should be considered. In contrast, we recently reported a syndrome of joint hyperlaxity, easy bruising, pelvic organs prolapses, premature rupture of the membranes and rectal bleeding associated with a non-glycine sequence variant of the *COL3A1 *gene (P435T) [54]. Whether non-glycine mutations of the *COL3A1 *gene may be responsible for a phenotype different from EDS type IV warrants further studies [54].

## Management including treatment

In the absence of specific treatment for EDS type IV, medical intervention should be focused on symptomatic treatment, prophylactic measures and genetic counselling. Patients should be advised to carry with them a letter or a card (such as the European Ehlers-Danlos syndrome passport) indicating the nature of their illness, the vascular or digestive complications to which they are at risk, their blood group and the contact details of a medical practitioner [5]. Intense physical activity, scuba diving and violent sports are inadvisable. Various medications such as acetylsalicylic acid, clopidogrel and/or antivitamin K drugs interfere with platelets functions or coagulation and should therefore be avoided.

Arterial, digestive or uterine complications require immediate hospitalisation, observation in an intensive care unit and sometimes surgery [24]. Arteriograms and endoscopies are contraindicated in principle. Conservative approach is usually recommended when caring for a vascular complication in a patient suffering from EDS type IV [24]. When the vital prognosis is not at stake, therapeutic abstention with close monitoring is indeed preferable to unjustified surgery [24].

Surgery may, however, be required urgently to treat potentially fatal complications such as uncontrolled haemorrhage or a very large or rapidly-expanding aneurysm [8,24]. To optimise the chances of success, the surgeon should be informed of the diagnosis before beginning surgery. Surgical precautions include delicate and atraumatic handling of tissues. The surgeon must choose the least complex and most direct repair technique possible [16]. The arterial ligature is an excellent choice when it does not compromise the bloody supply of an organ [24]. Simple arterial repairs have been successfully carried out in some cases [8]. Serious arterial complications require arterial reconstruction with prosthetic material. Anastomoses should not be carried out with tension but strengthened by Teflon *pledgets*. Despite these precautions, a number of patients develop post-operative haemorrhagic complications, as well as problems relating to anastomosis of the prosthetic graft [8]. At present, the information on the use of stents to treat vascular complications of EDS type IV is insufficient. The risk of arterial rupture distantly from the point of puncture is high. In all cases, it is imperative the post-operative monitoring to be prolonged and the post-operative checks by non-invasive imaging techniques (scanners) repeated [24]. It is essential that patients suffering from EDS type IV are not offered surgical procedures that are unessential, such as stripping of varicose veins [4,55].

Pregnant women with vascular EDS should be considered at risk and receive special care [32,56,57].

## Genetic counselling

Once the diagnosis has been confirmed, the opinion of a geneticist should be sought and family screening carried out. EDS type IV is a monogenic disorder, of autosomal dominant transmission [5]. Patients affected have a 50% risk of transmitting the disease to each of their children. The rate of *de novo *mutations is high and sporadic cases account for about half of all cases of EDS type IV. The hypothesis of recessive autosomal transmission of EDS type, still proposed in the eleventh edition of the hereditary monogenic disorders catalogue (OMIM), should be dismissed [5,7].

## Prenatal diagnosis

Molecular prenatal diagnosis can be considered for families where the mutation is known. Choriocentesis or amniocentesis entail, in theory, risks linked to the obstetric procedure in couples where the woman suffers from EDS type IV. Artificial insemination with donor sperm (when the patient is male) and adoption are other options to discuss with the couple during genetic counselling.

## Research prospects

The value of long-term beta blocker treatment (celiprolol) to prevent vascular complications in EDS type IV [58] is currently the subject of a controlled clinical trial (ClinicalTrials.gov Identifier: NCT00190411). However, its statistical analysis should focus on verifying the definite absence of methodological bias which the inclusion of patients with erroneous diagnosis of EDS type IV would constitute.
